# Appropriate Timing of Coronary Artery Bypass Graft Surgery for Acute Myocardial Infarction Patients: A Meta-Analysis

**DOI:** 10.3389/fcvm.2022.794925

**Published:** 2022-03-28

**Authors:** Qianlei Lang, Chaoyi Qin, Wei Meng

**Affiliations:** Department of Cardiovascular Surgery, West China Hospital, Sichuan University, Chengdu, China

**Keywords:** myocardial infarction, coronary artery bypass graft, early surgery, late surgery, meta-analysis

## Abstract

**Background:**

Currently, percutaneous coronary intervention (PCI) and coronary artery bypass grafting (CABG) are commonly used in the treatment of coronary atherosclerotic heart disease. But the optimal timing for CABG after acute myocardial infarction (AMI) is still controversial. The purpose of this article was to evaluate the optimal timing for CABG in AMI.

**Methods:**

We searched the PubMed, Embase, and Cochrane library databases for documents that met the requirements. The primary outcome was in-hospital mortality. The secondary outcomes were perioperative myocardial infarction (MI) incidence and cerebrovascular accident incidence.

**Results:**

The search strategy produced 1,742 studies, of which 19 studies (including data from 113,984 participants) were included in our analysis. In total, 14 studies compared CABG within 24 h with CABG late 24 h after AMI and five studies compared CABG within 48 h with CABG late 48 h after AMI. The OR of in-hospital mortality between early 24 h CABG and late 24 h CABG group was 2.65 (95%CI: 1.96 to 3.58; *P* < 0.00001). In the undefined ST segment elevation myocardial infarction (STEMI)/non-ST segment elevation myocardial infarction (NSTEMI) subgroup, the mortality in the early 24 h CABG group (OR: 3.88; 95%CI: 2.69 to 5.60; *P* < 0.00001) was significantly higher than the late 24 h CABG group. Similarly, in the STEMI subgroup, the mortality in the early 24 h CABG group (OR: 2.62; 95% CI: 1.58 to 4.35; *P* = 0.0002) was significantly higher than that in the late 24 h CABG group. However, the mortality of the early 24 h CABG group (OR: 1.24; 95%CI: 0.83 to 1.85; *P* = 0.29) was not significantly different from that of the late 24 h CABG group in the NSTEMI group. The OR of in-hospital mortality between early 48 h CABG and late 48 h CABG group was 1.91 (95%CI: 1.11 to 3.29; *P* = 0.02). In the undefined STEMI/NSTEMI subgroup, the mortality in the early 48 h CABG group (OR: 2.84; 95%CI: 1.31 to 6.14; *P* < 0.00001) was higher than the late 48 h CABG group. The OR of perioperative MI and cerebrovascular accident between early CABG and late CABG group were 1.38 (95%CI: 0.41 to 4.72; *P* = 0.60) and 1.31 (95%CI: 0.72 to 2.39; *P* = 0.38), respectively.

**Conclusion:**

The risk of early CABG could be higher in STEMI patients, and CABG should be delayed until 24 h later as far as possible. However, the timing of CABG does not affect mortality in NSTEMI patients. There was no statistical difference in perioperative MI and cerebrovascular accidents between early and late CABG.

## Introduction

Coronary heart disease (CHD), which is the main cause of death in middle-aged and elderly people, can lead to angina pectoris, myocardial infarction, and ischemic heart failure. Percutaneous coronary intervention (PCI) and coronary artery bypass grafting (CABG) can reconstruct adequate blood supply in myocardial blood supply areas caused by severe coronary artery stenosis (1). Although PCI has become the main intervention method for acute myocardial infarction (AMI), CABG is still a safe and feasible choice for patients with acute coronary syndrome (ACS). Moreover, it is an appropriate treatment for PCI failure, severe multivessel disease, or diabetes mellitus (2). CABG can obtain complete revascularization earlier and minimize cardiac ischemia.

However, the optimal timing for CABG is still controversial. Most of the literature does not define early CABG clearly, and the statistics were based more on the CABG time limit of 24 or 48 h. Some previous studies have shown that the mortality was higher in the early CABG (within 1 day, 2 days, or 1 week) group. Delayed CABG surgery was recommended in patients with AMI to reduce mortality. However, other research studies (3–9) showed that the timing of CABG did not affect the mortality of patients with ST segment elevation myocardial infarction (STEMI) or non-ST segment elevation myocardial infarction (NSTEMI). Therefore, the purpose of this study was to compare the mortality of early CABG (within 24 or 48 h after AMI) with that of late CABG for the optimal timing of CABG in patients with AMI, so as to better practice in the clinical work.

## Methods

### Publication Search

Two trained researchers independently searched articles in PubMed, Embase, and Cochrane library databases for suitable studies. The search form is [Myocardial Infarction (MeSH Terms)] OR [Infarction, Infarction (title/Abstract)] OR [Infarctions, Myocardial (title/Abstract)] OR [Myocardial infants (title/Abstract)] OR [Cardiovascular Stroke (title/Abstract)] OR [Cardiovascular Strokes (title/Abstract)] [Stroke, Cardiovascular (title/Abstract)] OR [Strokes, Cardiovascular (title/OR)] OR (Myocardial Infarctt) [Cardiovascular Stroke (title/Abstract)] OR [Cardiovascular Stroke (title/Abstract)] [Cardiovascular (title/Abstract)] [Myocardial (title/Abstract)] OR [Infarcts, Infarcts (title/Abstract)] OR [Myocardial infants (title/Abstract)] OR [Heart targets (title/Abstract)] OR [Heart targets (title/Abstract)] AND [coronary artery bypass (MeSH Terms)] AND [coronary artery bypass (MeSH Terms)] OR (Artery Bypass, (title/Abstract)] OR [Artery Bypasses, (title/Abstract)] [Coronary artists (title/Abstract)] OR [Coronary Artery classes (title/Abstract)] OR [Coronary Artery Bypass surfaces (title/Abstract)] OR [Bypass, Coronary Artery (title/Abstract)] OR [Aortocoronary Bypass (title/Abstract)] OR [Aortocoronary Bypasses (title/Abstract)] OR [Bypass, Aortocoronary (title/Abstract)] OR [Bypasses, Aortocoronary (title/Abstract)] OR [Aortocoronary Bypass (title/Abstract)] OR [Aortocoronary Bypasses (title/Aortocoronary)] OR [Aortocoronary Bypasses (title/title)] OR [Aortocoronary Bypasses (title/title)] Coronary Artery Bypass [Coronary surfaces (title/Abstract)] OR [Bypass, Coronary Art (title/Abstract)] OR [Aortocoronary Bypass (title/Abstract)] [Coronary artifact (title/Abstract)] OR [Coronary Artery Bypass grafting (title/Abstract)] OR [CABG (title/Abstract)] AND [time (Title/Abstract)] OR [early surgery (title/Abstract)] OR [late surgery (Title/Abstract)]. The purpose of this search strategy is to include the effects of early 24 or 48 h CABG and late 24 or 48 h CABG on in-hospital mortality in AMI. In addition, we also manually searched and supplemented the relevant literature.

### Study Selection and Data Extraction

The references were examined independently by two researchers, and the criteria were as follows: (1) population: patients undergoing CABG; (2) intervention: CABG in the early stage of AMI (<24 and <48 h); (3) comparative intervention: late CABG (>24 and >48 h); (4) results: the primary outcome included in-hospital mortality and the secondary outcome involved perioperative MI and cerebrovascular accident; and (5) study design: clinical-controlled trial. The exclusion criteria were as follows: (1) PCI for patients; (2) overlapping population; and (3) pediatric studies. The two researchers conducted an independent evaluation of the selected study and finally reached a consensus with a third researcher to resolve the final differences.

Two authors independently extracted data from the research and analyzed it. The included research should meet the criteria. The primary outcome was in-hospital mortality. The secondary outcomes were perioperative MI incidence and cerebrovascular accident incidence.

### Statistical Analysis

We evaluated the difference in in-hospital mortality between the early CABG group and the late CABG group. The types of myocardial infarction were divided into STEMI and NSTEMI and analyzed by subgroup analysis. The confidence intervals of odds ratio (OR) and 95%CI are used as summary statistics. Statistical heterogeneity is summarized by I^2^ statistics. The fixed-effect model or the random-effect model was selected according to heterogeneity. The overall effect was determined by the *Z*-test, Review Manager, version5.3 (The Nordic Cochrane Centre, Copenhagen, Denmark).

## Results

### Characteristics

**FIGURE 1:**
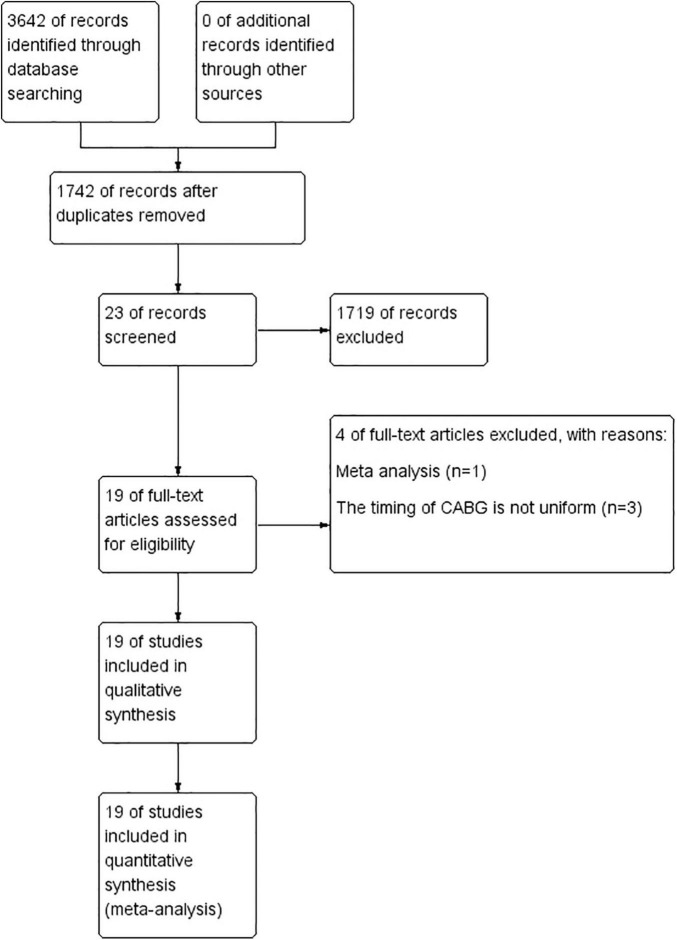
Study selection flow diagram.

**FIGURE 2:**
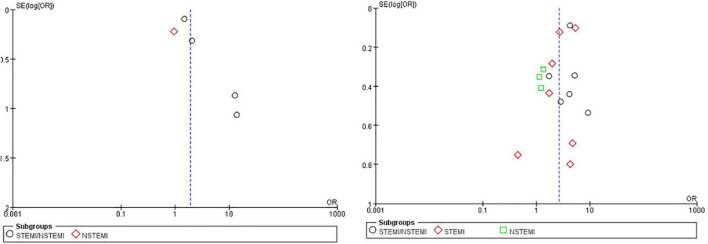
The funnel plot for included studies. The funnel plot was symmetrical which meant no significant publication bias. The former compared early 48 h CABG with late 48 h CABG, the latter compared early 24 h CABG with late 24 h CABG.

The search strategy produced 1,742 studies, of which 19 (data from 113,984 participants) were included in our analysis (Table 1). The Preferred Reporting Items for Systematic Reviews and Meta-Analyses (PRISMA) flow chart provided detailed descriptions of publication screening and reasons for exclusion are shown in Figure 1. There were 6 prospective studies (3–5, 8, 10, 11) and 13 retrospective studies (6, 7, 9, 12–21). A total of 7 studies (7–9, 11, 16, 18, 21) were evaluated in the STEMI population, 4 studies (5, 6, 8, 9) were evaluated in the NSTEMI population, and 10 studies (3, 4, 10, 12–15, 17, 19, 20) were evaluated in undefined STEMI/NSTEMI population. Two studies (8, 9) analyzed the data from patients of both STEMI and NSTEMI groups at the same time. We evaluated the quality of each literature according to Newcastle–Ottawa Scale (NOS). The funnel plot was symmetrical, which suggested no significant publication bias (Figure 2).

**TABLE 1:** Characteristics of the included studies.

References	Country	Patient number (n)	Type of MI	Time to CABG (day)	Age(years)	Gender (female), %	Patient number (n)	In hospital death (n)	In hospital mortality (%)	NOS
Braxton et al. (13)	United States	116	STEMI/NSTEMI	<2 3–5 6–42	NG	55 (4) 23 (3) 27 (26)	7 13 96	3 1 5	42.9 7.7 5.2	6
Creswell et al. (12)	United States	2296	STEMI/NSTEMI	<0.25 0.25–2 2–14 14–42 >42	62.3 ± 12.0 65.0 ± 11.3 63.9 ± 10.7 65.1 ± 10.5 63.6 ± 10.0	NG	11 132 869 261 1023	1 11 45 17 30	9.1 8.3 5.2 6.5 2.9	6 6
Kaul et al. (3)	United States	642	STEMI/NSTEMI	<0.25 0.25–1 1–3 3–7 7–14 14–28	NG	NG	46 121 140 135 129 71	9 5 6 5 8 5	19.56 4.13 4.28 3.7 6.2 7	
Lee et al. (4)	United States	316	STEMI/NSTEMI	<1 1–7 8–21	NG	NG	37 125 154	8 4 4	26.0 3.2 2.6	7
Bana et al. (14)	India	123	STEMI/NSTEMI	<2 3–14 15–30	NG	NG	10 36 77	2 1 1	20 2.8 1.3	6
Lee et al. (15)	United States	44365	STEMI/NSTEMI	<0.25 0.25–1 >1	NG	NG	885 556 42924	104 53 1207	11.8 9.5 2.8	7
Lee et al. (16)	United States	32099	STEMI	<0.25 0.25–1 1–3 4–7 8–14 >15	NG	27.8 (157) 23.4 (78) 25.9 (45) 26.1 (788) 26.6 (1095) 24.2 (5594)	564 333 946 3021 4118 23117	80 46 75 115 119 624	14.2 13.8 7.9 3.8 2.9 2.7	8
Voisine et al. (10)	Spain	7219	STEMI/NSTEMI	<0.25 0.25–1 1–7 8–30 >30	NG	NG	26 51 313 917 5912	5 5 27 29 144	19.2 9.8 8.6 3.2 2.4	8
Thielmann et al. (11)	Germany	138	STEMI	<0.25 0.25–1 1–3 4–7 8–14	NG	NG	37 21 15 24 41	4 5 1 1 1	10.8 23.8 6.7 4.2 2.4	8
Weiss et al. (17)	United States	9476	STEMI/NSTEMI	<2 >2	66.6 68.6	NG	4676 4800	262 182	5.6 3.8	8
Parikh et al. (5)	United States	2647	NSTEMI	<2 >2	63.0 65.0	24.5 (202) 31.7 (578)	825 1822	30 69	3.6 3.8	8
Filizcan et al. (18)	Turkey	85	STEMI	<0.25 0.25–1 15–30	NG	NG	33 24 28	2 12 2	6.1 50.0 7.1	7
Assmann et al. (19)	Germany	1168	STEMI/NSTEMI	<0.25 0.25–1 2–3 4–10 11–20 21–30	66.3 ± 13.4 66.5 ± 12.5 68.4 ± 13.6 66.5 ± 13.2 66.2 ± 12.8 67.4 ± 13.2	27 (4) 28 (14) 23.2 (22) 24.8 (29) 271 (99) 26.9 (141)	14 51 96 116 366 525	2 5 8 5 8 10	14.8 10.2 8.8 4.2 2.3 2.0	8
Davierwala et al. (6)	Germany	758	NSTEMI	<1 1–3 3–21	68.0 ± 10.0 70.0 ± 10.0 70.0 ± 10.0	23.3 (31) 26.6 (51) 23.8 (103)	133 192 433	8 9 22	6.0 4.7 5.1	8
Khan et al. (7)	United States	184	STEMI	<1 >1	63.0 ± 12.0 66.1 ± 11.0	25 (26) 33 (26)	105 79	19 9	18.1 11.4	8
Nichols et al. (20)	United States	3060	STEMI/NSTEMI	<1 1–2 3–7 8–21	NG	23.2 (23) 27.6 (102) 24 (472) 26.7 (167)	99 369 1966 626	5 7 33 14	5.0 2.0 2.0 2.0	8
Liakopoulos et al. (8)	Germany	1836	STEMI/NSTEMI	<1 1–3 >3	NG	NG	369 434 468	47 32 36	12.7 7.4 7.7	7
Lemaire et al. (21)	United States	5963	STEMI	<1 2–3 4–7	62.8 ± 11.6 63.2 ± 11.0 63.2 ± 10.8	24 (408) 23.3 (501) 22.3 (471)	1697 2154 2112	139 75 61	8.0 4.0 3.0	8
Bianco et al. (9)	United States	2058	STEMI/NSTEMI	<1 >1	66.0 66.0	26.39 (76) 29.31 (519)	288 1770	12 81	4.1 4.6	8

*CABG, coronary artery bypass graft; MI, myocardial infarction; NG, not given; NOS, Newcastle–Ottawa Scale; OR, odds ratio; STEMI, ST segment elevation myocardial infarction; NSTEMI, non-ST segment elevation myocardial infarction.*

### Primary Outcome

#### Early 24 h vs. Late 24 h Coronary Artery Bypass Grafting

We used in-hospital mortality as the primary outcome. A total of 14 studies were included, including 99,326 patients. Overall, in the early 24 h CABG group, in-hospital mortality was 4.0–24.6% and the average in-hospital mortality rate was 10.5% (575/5490). In the late 24 h CABG group, the in-hospital mortality was 1.8–11.4% and the average in-hospital mortality was 3.0% (2788/93836). The OR of in-hospital mortality between early 24 h CABG and late 24 h CABG group was 2.65 (95%CI: 1.96 to 3.58; *Z* = 6.35; I^2^ = 81%; *P* < 0.00001).

**FIGURE 3:**
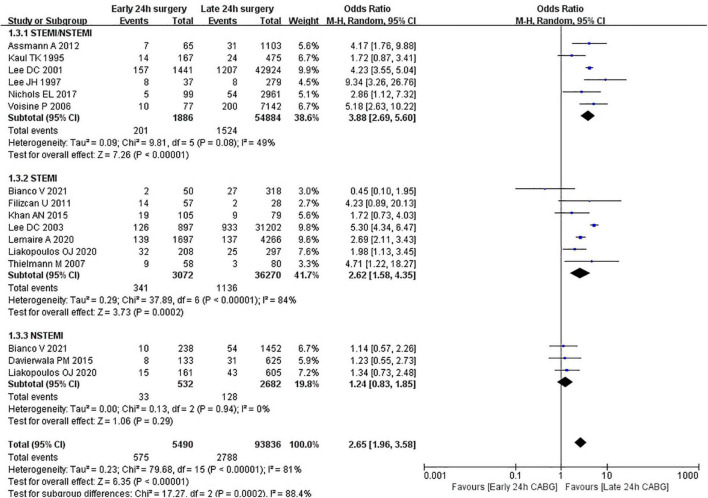
In-hospital mortality in early 24 h CABG groups vs. late 24 h CABG groups.

In addition, we divided the entire population into STEMI subgroup, NSTEMI subgroup, and undefined STEMI/NSTEMI subgroup. Subgroup analyses showed that in SETMI and undefined STEMI/NSTEMI subgroups, the mortalities in the early 24 h CABG group were significantly higher than those in the late 24 h CABG group (Figure 3). However, the heterogeneity of the STEMI group is very high. In the STEMI group, after excluding a study (16), we found that heterogeneity decreased significantly, and the conclusion was still consistent. However, in the NSTEMI subgroup, there was no significant difference in the mortality between the early 24 h CABG group and the late 24 h CABG group (OR: 1.24; 95%CI: 0.83 to 1.85; I^2^ = 0%; *Z* = 1.06; *P* = 0.29). The early 24 h CABG was associated with increased mortality except for the NSTEMI population.

#### Early 48 h vs. Late 48 h Coronary Artery Bypass Grafting

**FIGURE 4:**
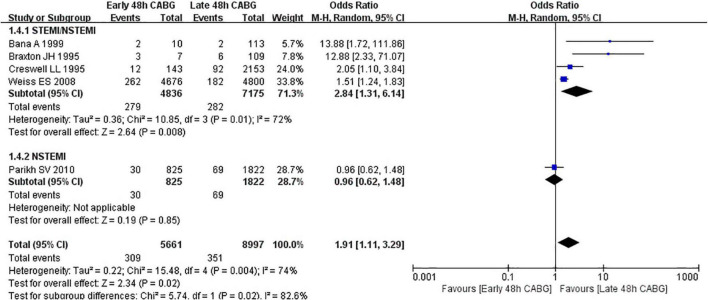
In-hospital mortality in early 48 h CABG groups vs. late 48 h CABG groups.

A total of 5 studies were included, including 14,658 patients. In the early 48 h CABG group, the in-hospital mortality was from 3.6 to 42.9%, and the average in-hospital mortality was 5.5% (309/5661). In the late 48 h CABG group, the in-hospital mortality was from 1.8 to 5.5%, and the average in-hospital mortality was 3.9% (351/8997). The OR of in-hospital mortality between the early 48 h CABG and the late 48 h CABG group was 1.91 (95%CI: 1.11 to 3.29; *Z* = 2.34; I^2^ = 74%; *P* = 0.02). In the undefined STEMI/NSTEMI subgroup, the mortality in the early 48 h CABG group (OR: 2.84; 95%CI: 1.31 to 6.14; *Z* = 2.64; I^2^ = 72%; *P* < 0.00001) was higher than that in the late 48 h CABG group. In the NSTEMI subgroup, only one study was included, and heterogeneity could not be calculated, with no difference in mortality between the early 48 h CABG and the late 48 h CABG group (OR: 0.96; 95%CI: 0.62 to 1.48; *Z* = 0.19; *P* = 0.85) (Figure 4).

### Secondary Outcomes

**FIGURE 5:**
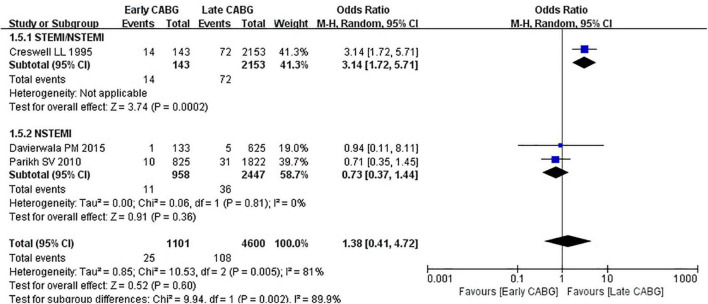
Perioperative myocardial infarction in early CABG groups vs. late CABG groups.

**FIGURE 6:**
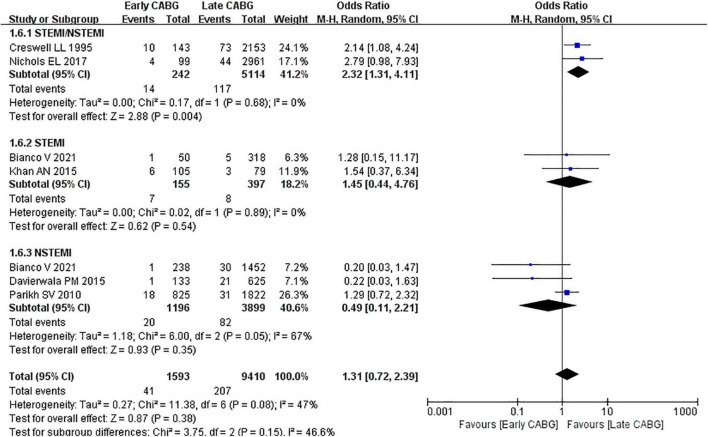
Cerebrovascular accident in early CABG groups vs. late CABG groups.

Perioperative MI and cerebrovascular accident were selected as secondary outcomes. The OR of perioperative MI incidence between early 24 or 48 h CABG and late 24 or 48 h CABG group was 1.38 (95%CI: 0.41 to 4.72; *Z* = 0.52; I^2^ = 81%; *P* = 0.60). In the NSTEMI subgroup, there was no significant difference in the perioperative MI between the early 24 or 48 h CABG group (OR:0.73; 95%CI: 0.37 to 1.44; *Z* = 0.91; I^2^ = 0%; *P* = 0.36) and the late 24 or 48 h CABG group. In the undefined STEMI/NSTEMI subgroup, heterogeneity could not be calculated because only one article was included. The OR of cerebrovascular accident incidence between early 24 or 48 h CABG and late 24 or 48 h CABG group was 1.31 (95%CI: 0.72 to 2.39; *Z* = 0.87; I^2^ = 47%; *P* = 0.38). In the undefined STEMI/NSTEMI subgroup, the OR was 2.32 (95%CI: 1.31 to 4.11; *Z* = 2.88; I^2^ = 0%; *P* = 0.04). In the STEMI subgroup, the OR was 1.45 (95%CI: 0.44 to 4.76; *Z* = 0.62; I^2^ = 0%; *P* = 0.54). In the NSTEMI subgroup, the OR was 0.49 (95%CI: 0.11 to 2.21; *Z* = 0.93; I^2^ = 67%; *P* = 0.35) (Figures 5, 6).

## Discussion

The optimal CABG time of patients with AMI is still a matter of debate. The results of a previous meta-analysis (22) showed that early CABG (within 24 or 48 h)after AMI increased patient mortality. However, only insufficient data for NSTEMI patients was available. Moreover, some of the recent studies we included contradicted the previous conclusion. In this article, patients with different types of myocardial infarction were divided into early 24 or 48 h CABG group and late 24 or 48 h CABG group. In conclusion, in the STEMI group, early CABG was associated with higher mortality, while in the NSTEMI group, the timing of CABG surgery did not affect the mortality of patients.

Some professionals recommended avoiding emergency CABG surgery for patients with STEMI because of the higher incidence of complications and mortality. Studies have shown that early coronary artery revascularization may increase the risk of death. If there was no absolute indication for emergency surgical intervention, such as structural complications and persistent ischemia, delayed surgery should be considered (8, 11, 16, 21). Additionally, other studies have identified early or emergency CABG as predictors of higher mortality (15, 17). On the contrary, studies (7, 9, 18) also showed that the timing of CABG surgery did not affect the mortality of patients with STEMI. It is worth noting that Bianco V et al. (9) conducted a large single-center retrospective study, which adjusted the baseline characteristics of patients in the early CABG group and the late CABG group by tendency score. Previous studies failed to identify the timing of CABG as an independent predictor of mortality (3, 23, 24).

There were several possibilities contributing to these contradictory conclusions: first, serum C-reactive protein (CRP), a marker of an acute inflammatory response, has been reported to rise sharply after transmural AMI, and it plateaued on day 3 after infarction. Furthermore, this peak level was a strong indicator of outcome after a first transmural myocardial infarction (25, 26). At the elevated stage of CRP, early surgical revascularization after AMI might further enhance this systemic inflammatory response and affect the prognosis because CABG was known to cause an increase in serum CRP level with or without cardiopulmonary bypass (27). Second, each surgeon and hospital may use different protocols and standards related to surgical techniques, cardiopulmonary bypass, and cardiac cardioplegia. Third, patients who need early CABG have significantly more complications, and surgeons have to perform immediately. Meanwhile, some patients in critical condition who could not afford early CABG were able to choose assisted circulation, such as cardiopulmonary bypass (CPB), to maintain hemodynamics, and then followed the treatment of CABG to obtain personalized and patient-friendly treatment strategies. On the other hand, some critical patients who were eligible for early CABG but did not undergo surgery for other reasons should be compared to other patients in the early CABG group, which was more meaningful. Although, some studies have shown that early CABG increased mortality in patients with STEMI, the optimal timing of CABG after AMI has not been determined. It is necessary to carry out appropriate effective RCT to determine whether early CABG actually increases patient mortality.

The incidence of NSTEMI has increased significantly compared to STEMI (28). Due to the progress of myocardial protection and mechanical support technology and the improvement of anesthesia and perioperative management in patients undergoing cardiac surgery, surgical intervention played an important role in the treatment of all these clinical conditions. CABG was used as a treatment choice for patients with NSTEMI. For NSTEMI patients, some researchers believed that the timing of CABG surgery after AMI did not affect the mortality of patients. Although the results of the study showed that there was no significant difference between early and late CABG, delayed surgical intervention in NSTEMI patients may lead to increased use of hospital resources, with little benefit to the patients (5). NSTEMI was characterized by non-transmural necrosis. Early blood supply reconstruction can prevent progression into transmural necrosis, limiting ventricular remodeling and maintaining left ventricular function.

In summary, the current literature comparing the timing of CABG following MI is at moderate to serious risk of bias due to patient selection and confounding (29). We also need further RCT to provide best practices for the best timing of CABG after AMI, especially for patients with STEMI, and we need to further study whether early CABG actually has a worse impact on patients in the future.

### Study Limitations

First, the included literature studies were retrospective, lacking relevant RCTs. A large number of multicenter RCTs were needed to prove whether early CABG actually increased the mortality of patients with AMI. Second, the undefined NSTEMI/STEMI subgroup had a large heterogeneity, which required further differentiation of patients with myocardial infarction type. Third, this meta-analysis set up two time-points, 24 and 48 h. Due to the lack of relevant original data, we could not objectively compare and judge which time point was better. Finally, there were pieces of literature containing secondary outcomes, and there was insufficient data to compare the secondary outcomes of early CABG and late CABG.

## Conclusion

In conclusion, patients with STEMI who underwent early CABG after AMI showed increased mortality. However, the timing of CABG did not affect mortality in NSTEMI patients. Meanwhile, there was no statistical difference in perioperative MI and cerebrovascular accident between early and late CABG. It was worth noting that the OR of mortality was progressively seen to be decreasing with the timing of CABG (2.65 at 24 h, 1.91 at 48 h). Actually, it is necessary to carry out appropriate effective RCTs to evaluate the results.

## Data Availability Statement

The original contributions presented in the study are included in the article/supplementary material, further inquiries can be directed to the corresponding author.

## Author Contributions

QL and CQ were responsible for retrieving literature, extracting, and analyzing the data. WM was responsible for the supervision and guidance. All authors made important contributions to the manuscript.

## Conflict of Interest

The authors declare that the research was conducted in the absence of any commercial or financial relationships that could be construed as a potential conflict of interest.

## Publisher’s Note

All claims expressed in this article are solely those of the authors and do not necessarily represent those of their affiliated organizations, or those of the publisher, the editors and the reviewers. Any product that may be evaluated in this article, or claim that may be made by its manufacturer, is not guaranteed or endorsed by the publisher.
